# Cardiogenetics, 25 years a growing subspecialism

**DOI:** 10.1007/s12471-020-01444-8

**Published:** 2020-08-11

**Authors:** A. A. M. Wilde, E. Nannenberg, C. van der Werf

**Affiliations:** 1 Department of Clinical and Experimental Cardiology, Heart Center, Amsterdam Cardiovascular Sciences, Amsterdam UMC, University of Amsterdam, Amsterdam, The Netherlands; 2 Department of Clinical Genetics, Amsterdam UMC, University of Amsterdam, Amsterdam, The Netherlands

**Keywords:** Genetics, Death, Sudden, Cardiomyopathies, Channelopathies

## Abstract

The cardiology and clinical genetics subspecialty of cardiogenetics has experienced a tremendous growth in the past 25 years. This review discusses examples of the progress that has been made as well as new challenges that have arisen within this field, with special focus on the Netherlands. A significant number of Dutch founder mutations, i.e. mutations shared by a number of individuals who have a common origin and all share a unique chromosomal background on which the mutation occurred, have been identified and have provided unique insights into genotype-phenotype correlations in inherited arrhythmia syndromes and inherited cardiomyopathies.

Cardiological and genetic screening of family members of young victims of sudden cardiac death combined with genetic testing in the deceased individual have turned out to be rewarding. However, the interpretation of the results of genetic testing in this setting and in the setting of living patients with a (suspected) phenotype is now considered more challenging than previously anticipated, because the introduction of high-throughput sequencing technologies has resulted in the identification of a significant number of variants of unknown significance. Interpretation of genetic and clinical findings by experienced multidisciplinary teams are key to ensure a high quality of care to the patient and the family.

## Dutch contribution to the field

- Determination of the genetic basis of inherited arrhythmia syndromes, including the Identification of new genes, among which the first gene for idiopathic ventricular fibrillation and the oligogenic nature of Brugada syndrome.
- Determination of the best practice of genetic counselling for inherited cardiac disorders.
- Detailed description of genotype-phenotype relationships in different inherited cardiac disorders.
- Determination of genetic and non-genetic modifiers of specific phenotypes

## Introduction

Cardiogenetics in the Netherlands started as a subspecialty within cardiology and clinical genetics with the discovery of the first genes responsible for hypertrophic cardiomyopathy (HCM) and congenital long-QT syndrome (LQTS) in 1990 and 1995, respectively [1, 2]. In the Netherlands, cardiogenetics clinics, a joint venture between cardiologists and clinical geneticists, were initiated in those early years in Maastricht, Groningen, Utrecht and Amsterdam (Academic Medical Center), in the years thereafter followed by the other academic hospitals and some non-academic hospitals, with Tilburg and Alkmaar notably early. In 2001, around 600 patients were seen in these clinics, being nearly 5% of all patients evaluated at a department of clinical genetics at that time. In 2007, these numbers had risen to 2500 patients and 10%, respectively, and in 2015 to almost 5500 patients and 15%. This tremendous growth can be attributed to increasing awareness among cardiologists and clinical geneticists that sudden cardiac death (SCD) at young age, often one of the sequelae of an inherited cardiac disease, can be prevented by timely recognition and preventive treatment of the respective disease. In addition, expanding technical possibilities of DNA testing contributed to this exponential growth.

## Founder mutations

**Fig. 1:**
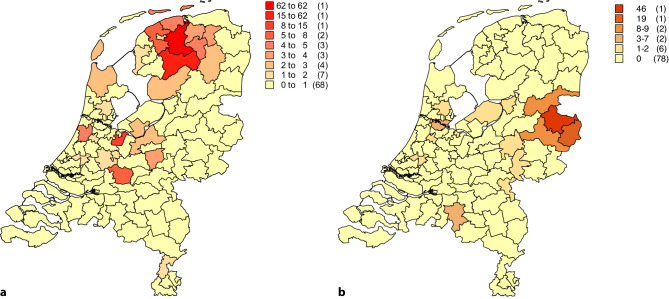
Two postal code maps of the Netherlands with two SCN5a mutations. In the left panel the SCN5a p.1795insD mutation is mapped and it is clear that the majority of patients reside in the north of the Netherlands (provinces of Friesland and Groningen). This mutation is a clear example of an overlap SCN5a phenotype (i.e. a phenotype with both characteristics of loss-of-function sodium channel activity and gain-of-function sodium channel activity). In the right panel the SCN5a c.2582-2583delTT is mapped and this mutation clearly originates from the east of the country (north of Enschede). This mutation is a pure loss-of-function variant (haploinsufficiency)

This development was paralleled by a series of publications on the various founder mutations in the different cardiological disease entities in the Netherlands in this journal, which were later merged in a booklet [3]. Founder mutations are mutations shared by a (large) number of individuals who have a common origin and all share a unique chromosomal background (haplotype) on which the mutation occurred. They appear to be frequent in the Netherlands, both in the cardiovascular domain [4–9], and in other domains [10]. Within the cardiovascular domain well-studied examples are HCM with three founder mutations being responsible for an estimated 40% of Dutch HCM patients [4, 11], the 1795insD mutation in the cardiac sodium channel (*SCN5A*) associated with a SCN5*A*-overlap syndrome (i.e., a disease entity with both gain of function (LQTS) and loss of function characteristics (progressive cardiac conduction disease and Brugada syndrome)) [7], a haplotype on chromosome 7 associated with idiopathic ventricular fibrillation [6], and the PLN Argdel14 mutation [8]. The high prevalence of founder mutations leads to region-specific prevalences of specific disease entities with the same underlying gene. The aforementioned *SCN5A* overlap mutation 1795insD is prevalent in the north of the Netherlands (Fig. 1), in the eastern part the *SCN5A* mutation c.2582-2583delTT is highly prevalent (associated with conduction abnormalities and occasionally Brugada syndrome, Fig. 1) and in the south the *SCN5A* mutation c.Phe1617del, also associated with an overlap syndrome, is highly prevalent [12].

## Family screening after sudden cardiac death in young individuals

Nowadays it is well accepted that active investigations in families where a young individual has died suddenly and unexpectedly can be rewarding. Initial studies, in the time domain where genetic screening consisted of a gene-by-gene approach, focused on clinical screening of first- and second-degree family members (ECG, exercise stress testing, transthoracic echocardiography and laboratory testing). In our hands a yield of finding a potential diagnosis was reached in 40% of families of individuals who had died suddenly under the age of 40 years, with the subsequent identification of almost nine presymptomatic disease carriers per family [13]. In an early sudden arrhythmic death syndrome cohort (i.e., sudden death in the absence of an identifiable cause after autopsy; SADS) the potential cause was identified by screening of family members in 32% of the families [14]. In larger series published in later years, it became evident that a lower age of the person who had died was associated with an increased likelihood of making a potential diagnosis (in up to 70% of families when the deceased victim was ≤10 years of age) and an increased likelihood of a familial electrical disease [15]. In sudden death victims between 30 and 50 years of age the likelihood of identifying a familial cardiomyopathy or ischaemic heart disease based on familial hypercholesterolaemia increased [15].

In early studies the incremental yield of genetic screening in the deceased individual (so-called ‘molecular autopsy’) was not particularly evident, but in later studies combined clinical screening of family and molecular autopsy reached a yield of almost 40% as well in a SADS cohort [16]. This does not distract from the great importance of autopsy after the (sudden) death of a young individual and that should include appropriate storage of DNA [17].

## Genetic testing

In general, the first attempt to determine the role of genetic testing for all inherited conditions associated with SCD was the 2011 consensus document endorsed by Heart Rhythm Society and the European Heart Rhythm Association [18]. For each condition, the impact of genetic testing was determined in three domains, i.e. the diagnostic, prognostic, and therapeutic domain. At that time LQTS scored highly positive in all of these domains whereas, for example, atrial fibrillation scored negatively in all domains [18]. Overall, the recommendations would not be so very different if a new consensus document were to be written today (Tab. 1). This was before the time of whole exome or whole genome screening, the role of which is yet to be defined, also in the setting of a molecular autopsy [19]. The ‘doubt’ relates to the issue of variants of uncertain significance (VUS, see below).

**Table 1:** The role of genetic testing for the index case in three categories (diagnostic, prognostic and therapeutic). The relative strength is indicated by the number of + with +++ as the strongest evidence and − as no evidence. The right column shows the yield of identifying a (putative) pathogenic mutation (^a^)

Disease	Diagnostic	Prognostic	Therapeutic	Yield^a^
LQTS	+++	+++	+++	±60–70%
CPVT	+++	+	–	±60%
SQTS	+	–	–	±30%
Brugada syndrome	+	+	+	±20–30%
CCD	+	–	–	Low
ERS	–	–	–	Low
Atrial fibrillation	–	–	–	Low
HCM	+++	+	+	±60%
DCM with CDD	+++	++	++	±70%
DCM without CCD	++	++	+	±30%
ARVC	++	++	++	±60%
RCM	+	–	–	?
NCCM	+	–	–	±30%

*LQTS* long QT syndrome, *CPVT* catecholaminergic polymorphic VT/VF, *SQTS* short QT syndrome, *CCD* cardiac conduction disease, *ERS* early repolarisation syndrome, *HCM* hypertrophic cardiomyopathy, *DCM* dilated cardiomyopathy, *ARVC* arrhythmogenic right ventricular cardiomyopathy, *RCM* restrictive cardiomyopathy, *NCCM* non-compaction cardiomyopathy

The benefit of early identification of affected family members obviously relates to the possibility to start timely treatment. Whether preventive treatment has proven efficacy in reducing the number of SCDs in different disease entities has not yet been shown for all diseases, but it is generally accepted that preventive treatment in many of these disorders is beneficial. Good examples are the TMEM43 mutations-related arrhythmogenic cardiomyopathy, highly prevalent in Newfoundland, Canada [20], and the DPP6-haplotype on chromosome 7 associated with idiopathic ventricular fibrillation [21]. In both conditions, prophylactic implantable cardioverter-defibrillator (ICD) implants, just on the basis of harbouring the genetic abnormality, have been demonstrated to save lives [20, 21]. The same is expected for presymptomatic pharmacological treatment of individuals with a pathogenic mutation associated with LQTS or catecholaminergic polymorphic ventricular tachycardia.

Identifying the underlying genetic substrate is also critical for treatment choices. Well-known examples to support this statement are LQTS, where in almost every aspect of the disease the underlying genotype is of importance, and in some subtypes of dilated cardiomyopathy. In LQTS, this includes the age of onset and the triggers of symptoms, the baseline ECG manifestation, the response of the QTc interval to exercise, and the mode of onset of the potential lethal arrhythmias [22]. Genotype-specific treatment is also pertinent although all subtypes respond well to β‑blockade, the cornerstone of the pharmacological treatment in this disease. However, the age where treatment should be started may differ among genotypes (LQT1 patients need treatment from birth onward whereas treatment in LQT2 and LQT3 patients can probably be delayed until puberty in the absence of prolonged QTc intervals at rest). Mexiletine has been shown to be very effective in LQT3 and probably also in LQT2, and potassium suppletion is particularly effective in LQT2. Avoidance of specific triggers is pertinent in a genotype-specific manner, i.e. unattended swimming in LQT1 and loud noises in LQT2. Examples in the cardiomyopathy field of genotype-specific treatment can be found in dilated cardiomyopathy, where LMNA or PLN mutation associated cardiomyopathies are associated with an ‘arrhythmogenic phenotype’ mandating early ICD implant, at left ventricular ejection fraction (LVEF) of ≤45%, instead of the usual LVEF ≤35% cut-off in other subtypes [23]. In other disease entities, such as Brugada syndrome of hypertrophic cardiomyopathy, the underlying genotype does not as yet impact on treatment choices.

It is important to emphasise that cardiogenetic evaluation after a sudden cardiac arrest or SCD is ideally performed in a multidisciplinary setting with involvement of dedicated (paediatric) cardiologists, clinical geneticists (or genetic counsellors), molecular geneticists, pathologists and psychosocial workers [17–19]. The coordination of all these specialists requires the formation of a multidisciplinary team with regular involvement of all these players [17–19].

An important task of this multidisciplinary team is ‘mutation calling’, i.e. the process of interpreting an identified variant. With the rapidly expanding gene panels (with regard to number of genes analysed), the number of VUSs is exponentially growing and as such is turning out to become the Achilles’ heel of molecular genetic testing. The presence of a VUS leaves the patient and his/her treating physician potentially in a place called ‘genetic purgatory’ [24]. Within the recent literature there are some devastating examples of what can happen after an incorrect interpretation of a variant-disease relationship [25]. With the combined expertise of molecular geneticists and cardiologists the VUS burden can be decreased, as has been recently shown in catecholaminergic polymorphic ventricular tachycardia [26]. At the same time, further expansion of gene panels should actually be avoided because of the accumulating evidence that previous gene assignments seem to be wrong or at least too premature. In a wide range of disease entities (e.g. HCM, Brugada syndrome, LQTS) a recent critical reanalysis of the assigned genes has downgraded many of them to limited evidence for pathogeneity [27–29]. This should actually lead to abandoning these genes from the relevant panels, which is not yet always the case in daily practice.

For some of the monogenic disorders it is becoming increasingly clear that they might be less ‘monogenic’ than initially thought. The paradigm for this statement seems Brugada syndrome where to date a pathogenic variant is identified in only 20 to 30% of the patients, the vast majority in the SCN5a gene [30]. A genome-wide association study in 2013 found that three loci in the genome associate with the signature ECG in Brugada syndrome patients. The more loci an individual patient accumulates, the more likely he or she (mostly he) will present with a type 1 Brugada ECG [31]. This points to an oligogenic or polygenic nature of this disease instead of a pure monogenic disease. It has also been shown that the same genetic factors underlie the variability in the response to sodium channel blockers [32]. Together with the baseline ECG and the family history the polygenic risk score (using the same three loci in the genome) predicts the development of a type 1 Brugada ECG during a sodium blocker challenge test [32]. It seems likely that the concept of an oligogenic or polygenic inheritance pattern may also be pertinent to other cardiogenetic disorders.

In more prevalent cardiovascular disease entities such as coronary artery disease, atrial fibrillation and diabetes mellitus type 2, the role of genetics is more limited but the cumulative effect of genetic factors (the ‘polygenic risk score’) has been shown to be very well able to identify an important subset of the population at risk [33]. It is to be expected that also for inherited cardiac diseases, these risk scores will be introduced and classify patients at risk in our daily clinical practice.

## Conclusions

In summary, cardiology and clinical genetics have witnessed a tremendous growth of the cardiogenetics subspecialty in the last two decades. It started approximately 25 years ago with monogenic (‘familial’) cases and with the associated family screening it has broadly introduced the concept of presymptomatic screening and treatment in the cardiovascular field. The genetic basis has also been shown to be important, and in some conditions even critical, for treatment decisions. Although we have made tremendous progress in unravelling the genetic background of inherited diseases in the last decades, the number of VUSs is exponentially growing and as such is turning out to become the Achilles’ heel of molecular genetic testing. Experienced multidisciplinary teams are required for proper interpretation of these genetic (and eventual clinical) findings and for further recommendations on treatment and family counselling.
